# Changes in smoking prevalence among first- and second-generation Turkish migrants in Germany – an analysis of the 2005 Microcensus

**DOI:** 10.1186/1475-9276-8-26

**Published:** 2009-07-20

**Authors:** Anna Reeske, Jacob Spallek, Oliver Razum

**Affiliations:** 1 University of Bielefeld, Department of Epidemiology & International Public Health, School of Public Health, P.O. Box 10 01 31, D-33501 Bielefeld, Germany; 2 Johannes Gutenberg – University Mainz, University Medical Center, Institute of Medical Biostatistics, Epidemiology and Informatics (IMBEI), Department of Epidemiology, Obere Zahlbacher Str. 69, D-55131 Mainz, Germany

## Abstract

**Background:**

Compared to the majority population of a host country, migrants tend to have different health risks and health behaviour. We have hypothesised that these differences diminish with time passed since migration. We tested this hypothesis by examining smoking behaviour among Turkish migrants and their children born in Germany (second-generation migrants), stratified by educational level and, for the first generation, length of residence.

**Methods:**

We estimated the prevalence of smoking based on the representative 2005 *Mikrozensus*, an annual survey including 1% of Germany's households. The 2005 Microcensus was the first to provide information that makes it possible to differentiate between first- and second-generation Turkish migrants. In total, 12,288 Turkish migrants and 421,635 native-born Germans were included in our study. The unit non-response is generally low (about 6% in 2005) because participation in the Microcensus is obligatory.

**Results:**

We found the prevalence of smoking in second-generation male Turkish migrants to be lower than in the first-generation group: 47.0% of first-generation Turkish migrants with a high level of education were smokers, in contrast to only 37.6% in the second generation. Within the German reference population, 29.9% were smokers. The percentage of Turkish women in our sample who smoked was generally smaller, but was not significantly lower in the second generation. In fact, the prevalence of smoking was highest among Turkish women of the second generation with a low level of education (40.9%), similar to younger second-generation German women with the same level of education.

**Conclusion:**

We present the first representative data on changes in the prevalence of a risk factor for many chronic diseases among Turkish migrants in Germany. Male Turkish migrants (with a high level of education) showed a decrease over the generations while smoking prevalences of female Turkish migrants increased. In the second generation, prevalences partly converged with those of the German reference population or were even higher. Our hypothesis – that migration can be interpreted as a "health transition" – was thus partly confirmed.

## Background

The prevalence of smoking, an important risk factor for cardiovascular diseases and several types of cancer, differs between countries. A Turkish-German study, which compared smoking prevalences in Turkey and Germany, has shown that smoking prevalence among men in Turkey is higher than in Germany [1]. WHO statistics show that in 2003, smoking prevalence among men was 49.4% in Turkey versus 37.1% in Germany, while smoking prevalence among women was more than 10% higher in Germany (17.6% vs. 30.5%) [2]. Turkish migrants to Germany thus migrate from a country with a high smoking prevalence among men and a low prevalence among women to a country with a lower smoking prevalence among men and a comparatively higher prevalence among women. In Germany, little is known about the development of smoking behaviour among migrants from Turkey after migration.

Compared to the majority population of a host country, migrants tend to have different health risks and show different health behaviour [3]. These differences are influenced by living conditions, e.g. dietary habits, access to health services, risk behaviour, and socioeconomic factors such as housing and working conditions (commonly subsumed under the term 'nurture'), genetic disposition ('nature'), and, finally, the process of migration itself, which can be associated with factors such as physiological and psychological stress [4]. The differences in health risks and resources between migrants and the native population change with the length of time passed since migration, and especially over generations, and it has been postulated, and partly shown, that migration can be described as a "health transition" [5]. This model holds that in the host country, migrants from low-income countries are faced with more advanced health care services, but also a different, 'Western', life style. Thus, while mortality and morbidity from causes such as infectious agents will decrease quickly thanks to better hygienic conditions and health services, the incidence of some chronic diseases, such as cancer and cardiovascular diseases, will increase due to changes in life style (e.g. diet, smoking behaviour, and physical activity). Depending on genetic makeup and latency periods, this increase may be slow. The model predicts that smoking prevalence among migrants from Turkey to Germany should change with increasing duration of residence.

Persons with migration experience (or with migrated parents) show a higher smoking prevalence than Germans without migration experience [6,7]. A German study found that the percentage of smokers among Turkish nationals (15–24 years old) increased with increasing duration of residence, especially among men [8].

International studies on smoking and migrants have reported partly high smoking prevalences in migrant populations [9,10] and differences in smoking prevalence between migrants and the native population [11-14]. A Dutch study analysed whether behavioural risk factors over two generations of Turkish migrants in the Netherlands converge towards that of the native Dutch population [15]. The authors found that smoking prevalence was highest in male first-generation migrants while the second generation converged to the lower prevalence of the Dutch reference population. Among women, there was no convergence over the generations: Smoking prevalences in the first generation and the Dutch reference population were similar while prevalences in the second generation were higher than either. Smoking prevalence was presented by generational status and sex but was not stratified for socioeconomic status and age.

Since the mid 1950s, Germany has been among the countries in Europe accepting the largest numbers of migrants. During the 1960s economic boom, Germany's government supported the hiring of workers from Mediterranean countries to meet the labour demand in Germany. From 1955 to 1968, agreements with several countries were signed, among them Turkey (1961). Young men, and, later, women from rural areas came to Germany as so-called *Gastarbeiter *('guest workers'). With the 1973 economic recession and oil crisis, recruitment of 'guest workers' was stopped. At this time, the number of 'guest workers' in Germany was 2.6 million [16]. In a second migrant wave during the 1980s, former 'guest workers' began to move their families to Germany. Thus, since the 1960s, the number of Turkish migrants has increased continuously. By the end of 1997, 62% of the Turkish residents had lived in Germany for more than 10 years [17]. Today, many former guest workers who migrated themselves – migrants of the first generation – are now the parents' generation. Thus, a large number of ethnic Turks who live in Germany were born in Germany, but have not migrated themselves. They are referred to as migrants of the second generation or persons with a Turkish 'migration background'.

In 2005, 15.3 million or 19% of Germany's population had a migration background. There were 10.4 million first-generation migrants and 4.9 million second-generation migrants [18]. Turkish nationals represented the largest foreign population group in Germany (26.1%) [19].

In this study we tested the model of migration as a "health transition" by analysing smoking patterns. We examined (I) differences in smoking prevalence between first-generation Turkish migrants and their children born in Germany (second generation) as well as (II) possible trends among first-generation Turkish migrants with increasing time passed since migration. Our aim was to investigate whether the smoking patterns of Turkish migrants who were longer in the country, or who belonged to the second generation, were more similar to that of the German population.

The present study is the first to use Microcensus data to differentiate between first- and second-generation migrants, stratified by educational level, and also uses information on length of residence.

## Methods

### Data set

The prevalence of smoking was estimated on the basis of the German 2005 *Mikrozensus*. The Microcensus is a representative, country-wide, annual survey conducted by the *Statistisches Bundesamt *(Federal Statistical Office) in Germany. In total, 1% of all households in Germany are included each year. This means 380,000 households, or 820,000 persons, are included. We used a Scientific Use File, which is a 70% sub-sample of the 1% survey. Households are included on the basis of 'partial rotation', meaning each of the chosen households is included for four years, and every year, 25% of the included households leave the survey and are replaced by new ones [18]. Participation in the Microcensus is obligatory. It comprises questions that are asked annually, e.g. questions about demography, economic and social conditions. Every fourth year, a health module is included. These additional questions can be answered voluntarily, while the annual programme is obligatory. For the present analysis, we were interested in the questions about smoking habits, which are part of the health module.

The preferred interviewing method is a face-to-face interview conducted by trained interviewers. Answers are directly entered into data collection software using mobile computers (laptops). Approximately 12% of the respondents fill in their questionnaires by themselves. Among young people aged 15–19, there is a high proportion of proxy interviews (75%) and a moderate proportion of 25–30% for older people [20]. If necessary, the questionnaires for Turkish respondents are translated into their mother tongue to ensure they can understand the questions.

The unit non-response is generally low (about 6% in 2005) because it is obligatory to participate in the Microcensus. The item non-response is mostly under 10% [20]. Information about differences in the response rate between Turkish migrants and native-born Germans was not available because we used a more detailed definition of Turkish migrants than was employed earlier (see below). The Turkish migrants in this study are most likely representative of the Turkish population in Germany as a whole, because the questionnaire is translated, answering is obligatory, the sample size is large enough and the data collection method used by the Federal Statistical Office is well established.

### Definition of the study subgroups

In 2005, the Microcensus included an extended list of questions about migration. Before 2005, it had been possible to define migration background only by nationality as a crude proxy. Since 2005, the Microcensus has included detailed questions on migrant status. Thus, we were able to differentiate between first- and second-generation migrants for the first time. In this study, we defined first- and second-generation Turkish migrants by their country of birth (Table 1). More specifically, we classified persons as first-generation Turkish migrants when they were not born in Germany (and had the Turkish nationality or had had it before naturalisation) while the second generation was defined as born in Germany (and having the Turkish nationality or having had it before naturalisation). People of the second generation who acquired German citizenship by birth were also defined as second-generation migrants if at least one of their parents was Turkish-born (regardless of whether that parent was later naturalised). As reference population, we used native-born Germans. This group includes every respondent who possessed German citizenship and both of whose parents were native Germans.

**Table 1:** Variables used for the definition of first- and second-generation Turkish migrants

**first generation**			**second generation**				
		**not naturalised**	**naturalized**				
			
**not naturalised**	**naturalized**		**1st possibility**	**2nd possibility**	**3rd possibility**	**4th possibility**

not born in Germany	not born in Germany	born in Germany	born in Germany	born in Germany	born in Germany	born in Germany
no German nationality	German nationality through naturalisation	no German nationality	German nationality through naturalisation	German nationality through naturalisation	German nationality through naturalisation	German nationality
Turkish nationality	Turkish nationality before naturalisation	Turkish nationality	Turkish nationality before naturalisation	mother/father had Turkish nationality before naturalisation	mother/father has Turkish nationality	Turkish nationality as second one

### Smoking

The question on the smoking status addressed all respondents older than 10 years. The wording is: "Are you currently a smoker?". The possible answers are 'regular', 'occasional' and 'no'. For estimating the prevalence of smoking, regular and occasional smokers were combined because the proportion of occasional smokers was very small. Furthermore, these two categories were not defined in the questionnaire and have a low discriminatory power because they are self-reported and not verified using biological markers.

### Level of education

Since it is known that smoking is associated with social status, we stratified the smoking prevalence for level of education, which was used as a proxy variable for social status. Level of education was dichotomised based on the highest school degree or vocational qualification in the German educational system, which does not have exact counterparts in most other countries. Roughly, we defined a low level of education as anything less than 10 years of school, or no vocational training below that required to fill a senior position in a given trade. All educational attainments higher than that were defined as a high level. For details, please see the footnote in Table 2. Respondents younger than 18 years were excluded from the analysis, because the final level of education cannot be defined for most people under the age of 18.

**Table 2:** Characteristics of the study population

	1^st ^generation by duration of residence						1^st ^generation (total)		2^nd ^generation		native-born Germans	
	0–15		16–31		≥ 32							
	n	%	n	%	n	%	n	%	n	%	n	%
*Gender and age group*												
**Males**	840		1223		1002		3510		1022		166522	
18–24	94	11.2	59	4.8	0	0.0	174	5.0	427	41.8	16883	10.1
25–44	684	81.4	876	71.6	186	18.6	1989	56.7	574	56.2	55026	33.0
45–64	59	7.0	266	21.7	589	58.8	1056	30.1	17	1.7	56359	33.8
65+	3	0.4	22	18.0	227	22.7	291	8.3	4	0.4	38254	23.0
**Females**	868		1370		687		3368		1005		183309	
18–24	186	21.4	67	4.9	0	0.0	294	8.7	460	45.8	16021	8.7
25–44	590	68.0	850	62.0	132	19.2	1799	53.4	518	51.5	54309	29.6
45–64	81	9.3	406	29.6	455	66.2	1090	32.4	24	2.4	57899	31.6
65+	11	1.3	47	3.4	100	14.6	185	5.5	3	0.3	55080	30.0
												
*Gender and education*												
**Males**	833		1215		993		3481		1019		165072	
High	244	29.3	267	22.0	153	15.4	747	21.5	422	41.2	91384	55.4
Low	589	70.7	948	78.0	840	84.6	2734	78.5	597	58.6	73688	44.6
**Females**	865		1360		683		3343		999		181114	
high	167	19.3	214	15.7	76	11.1	514	15.4	498	49.8	95148	52.5
low	698	80.7	1146	84.3	607	88.9	2829	84.6	501	50.2	85966	47.5

The precise cut-off point we chose between 'high' and 'low level of education' is in fact a peculiarity of the German three-tier secondary school system, where pupils are assigned to one of three types of schools after 4 years of primary school based on their primary school teachers' recommendations: the *Gymnasium*, which takes another 9 years of school (13 in all), and prepares for academic studies, the *Hauptschule*, which takes another 5 or 6 years (9 or 10 in all), and prepares for an apprenticeship for professions which might tentatively be categorised as blue-collar jobs, and, finally, the *Realschule*, which, in a manner of speaking, takes the middle ground: it always lasts for another 6 years (10 in all) and even though it is not intended to prepare for academic studies either, the professions it prepares for can be tentatively categorised as white-collar jobs. An important difference is that the *Realschule *curriculum includes two foreign languages, as opposed to only one in the *Hauptschule*. We defined a *Realschule *degree, regardless of further education, as a high level of education. If a respondent had no degree or 'only' a *Hauptschule *degree, (s)he was categorised as having a low level of education but if (s)he completed later vocational training advanced enough to be qualified to formally train apprentices her-/himself (*Meister/Techniker(in) *etc.) it was categorised as high level of education.

### Analysis

In this descriptive study, the chi-square test was used for statistical testing of differences in smoking prevalences between first-generation migrants, second-generation migrants and the native-born Germans as well as differences between the three categories of duration of residence in the first generation. The significance level was set at 0.05. Analyses were stratified for sex, age, and level of education.

All statistical analyses were performed using SPSS version 15 (SPSS Inc., Chicago, IL, USA). Missing values were not imputed.

## Results

The 70% sub-sample of the 2005 Microcensus includes 477,239 persons. We identified 7,205 Turkish migrants of the first generation (1.5% of the total), 5,083 Turkish migrants of the second generation (1.1% of the total) and 421,635 native-born Germans (88.3% of the total). The remaining 9.1% were persons with a non-Turkish migration background, and this group was excluded from our sample.

Of all respondents (n = 477,239), nearly 20% smoked cigarettes regularly or occasionally. There were 57.4% non-smokers, 8.8% of respondents were under the age of ten, and 14.6% did not answer the question. In the study population (n = 433,923), which excluded any non-Turkish migrants, the proportion of smokers was 27.0%. The non-response concerning smoking status was 21.0% among first-generation Turkish migrants, 19.5% among the second generation and 15.6% among the native-born Germans.

Table 2 shows the demographics of the Turkish migrants and the native-born Germans. Turkish migrants are presented according to their generation and, for the first generation, length of residence.

### Males

There are statistically significant differences in the smoking prevalence between male Turkish migrants of the first and second generation and the German reference population (see table 3). We found a difference in smoking prevalence between the first and the second generation of male Turkish migrants with a high level of education: 47.0% among Turkish men in the first generation were smokers, as opposed to only 37.6% in the second generation. Within the German reference population, nearly 30.0% were smokers. In contrast, smoking prevalences among male Turkish migrants with a low level of education were higher in the second generation (first generation: 50.5%, second: 56.2%). Compared with the native-born Germans, first- and second-generation migrants showed considerably higher smoking prevalences in all age groups, except in the youngest age group (18–24 years). In addition, there is an obvious effect of the level of education, because the smoking prevalence among men with a low level is generally higher than among men with a high level of education (Table 3).

**Table 3:** Number of smokers and non-smokers (n) and smoking prevalence (%) (males)

	Age in years	1^st ^generation (total)		2^nd ^generation		native-born Germans		p-values^1^	1^st ^generation by duration of residence						
									0–15		16–31		≥ 32		p-values^2^
		n	%	n	%	n	%		n	%	n	%	n	%	
High level of education															
	18–24	38	42.1	141	27.7	10202	35.3	0.11	19	31.6	14	42.9	-	-	0.51
	25–44	418	**51.2**	192	**44.8**	31412	**35.4**	**<0.01**	184	53.3	140	50.7	51	47.1	0.72
	45–64	135	**39.3**	-*	-*	24871	**28.5**	**0.02**	6	50.0	56	41.1	58	41.4	0.91
	65+	20	20.0	0	0.0	10043	10.6	0.18	0	0.0	0	0.0	17	23.5	-
	total	611	**47.0**	335	**37.6**	76528	**29.9**	**<0.01**	209	51.2	210	47.6	126	41.3	0.21

Low level of education															
	18–24	90	**40.0**	207	**46.4**	3807	**60.4**	**<0.01**	51	39.2	31	35.5	-	-	0.74
	25–44	1202	**58.9**	270	**64.8**	13516	**50.9**	**<0.01**	378	60.8	576	57.5	110	64.5	0.30
	45–64	667	**44.4**	12	**41.7**	22689	**35.4**	**<0.01**	39	46.2	154	50.0	393	43.8	0.42
	65+	192	**24.0**	-*	-*	23783	**14.1**	**<0.01**	-*	-*	15	26.7	149	20.8	0.15
	total	2151	**50.5**	493	**56.2**	63795	**32.2**	**<0.01**	471	**57.3**	776	**54.5**	652	**42.0**	**<0.01**

-* = number smaller than 5

^1^p-values are the results of chi-square testing for differences between 1^st ^(total), 2^nd ^generation Turkish migrants, and native-born Germans

^2^p-values are the results of chi-square testing for differences between first-generation migrants with different durations of residence

There were also significant differences among first-generation migrants with different durations of residence (see table 3). Among male Turkish migrants with a high level of education, smoking prevalence decreased with increasing duration of residence, i.e. migrants who had lived in Germany for more than 31 years had the lowest smoking prevalence (41.3%) and approached the smoking prevalences of the second-generation migrants (37.6%) and the native-born Germans (29.9%).

Among men with a low level of education, smoking prevalence was also lowest among first-generation migrants who had lived in Germany for more than 31 years (except in the age group between 25 and 44), and was nearer to that in the German reference population. In the age group between 25 and 44, the first generation (≥32 years of residence) has a smoking prevalence comparable to that in the second generation.

### Females

In contrast to the Turkish male population, a smaller percentage of Turkish women in our sample smoked. Among Turkish women, the second generation had a higher smoking prevalence after age adjustment both among women with a high and with a low level of education. Among women with a high level of education, this difference was modest, i.e. between 31.6% (first) and 33.3% (second generation). At the same time, both the first and second generation exceeded the prevalence of the female native-born Germans (24.5%). The difference among Turkish females with a low level of education was even more obvious (first generation: 24.5%, second generation: 40.9%). In the youngest age group, the prevalence was highest among native-born Germans (53.4%), and between 25 and 44 years, it was similar between native-born Germans and second-generation migrants (Table 4).

**Table 4:** Number of smokers and non-smokers (n) and smoking prevalence (%) (females)

	Age in years	1^st ^generation (total)		2^nd ^generation		native-born Germans		p-values^1^	1^st ^generation by duration of residence						
									0–15		16–31		≥ 32		p-values^2^
		n	%	n	%	n	%		n	%	n	%	n	%	
High level of education															
	18–24	75	24.0	208	26.9	11086	31.4	0.15	39	**12.8**	31	**32.3**	-	-	**0.05**
	25–44	275	**32.7**	204	**39.7**	34282	**28.7**	**<0.01**	90	36.7	130	34.6	30	20.0	0.23
	45–64	66	**36.4**	0	**0.0**	24966	**22.2**	**0.01**	-*	-*	20	55.0	35	25.7	0.09
	65+	5	20.0	0	0.0	9784	8.0	0.32	0	0.0	-*	-*	-*	-*	0.51
	total	421	**31.6**	412	**33.3**	80118	**24.5**	**<0.01**	132	29.5	182	36.3	68	23.5	0.13

Low level of education															
	18–24	163	**21.5**	165	**33.9**	2258	**53.4**	**<0.01**	113	16.8	28	32.1	-	-	0.07
	25–44	1162	**32.4**	215	**48.8**	10195	**46.0**	**<0.01**	377	**28.6**	569	**33.9**	81	**44.4**	**0.02**
	45–64	778	**16.6**	21	**19.0**	24318	**26.6**	**<0.01**	63	14.3	311	15.1	312	18.3	0.50
	65+	134	6.0	-*	-*	37469	5.8	0.94	9	0.0	34	0.0	75	8.0	0.16
	total	2237	**24.5**	403	**40.9**	74240	**19.6**	**<0.01**	562	24.2	942	26.4	468	21.2	0.09

-* = number smaller than 5

^1^p-values are the results of chi-square testing for differences between 1^st ^(total), 2^nd ^generation Turkish migrants, and native-born Germans

^2^p-values are the results of chi-square testing for differences between first generation migrants with different durations of residence

In contrast to male Turkish migrants, smoking prevalence did not decrease with increasing duration of residence in the first generation among female Turkish migrants with a high level of education; the prevalence was highest among females with 16–31 years of duration of residence (36.3%) and lowest among female migrants with the longest duration of residence (23.5%). But the number of female smokers in the first generation with a high level of education was too small to state a trend. Among the female first generation with a low level of education, smoking prevalence increased with increasing duration of residence in the age groups 25–44 and 45–64 years; also, the longer the residence, the more prevalences resembled those of the second generation and those of the second-generation German women.

## Discussion

We present the first representative data on the prevalence of smoking, a risk factor for cancer and cardiovascular diseases that are differentiated between first and second generation Turkish migrants and, for the first generation, broken down by duration of residence in Germany.

The model which interprets migration as a "health transition" was partly confirmed. Differences in smoking prevalence between migrants and the native population of a host country have been reported in several studies [6,7,12,14]. We also found differences between Turkish migrants and the native-born Germans. Male first-generation migrants from Turkey showed smoking prevalences higher than that of native-born Germans, but similar to the high, and, over time, only slightly decreasing smoking prevalences in Turkey [1,2]. In fact, the prevalence of smoking among female first-generation Turkish migrants in Germany seems to be higher than in Turkey, and partly even higher than the prevalence among native-born Germans.

In line with the findings of Hosper et al. [15], we found that trends of convergence in smoking prevalences differed between men and women. Our hypothesis that migration can be described as a health transition was confirmed for the case of men with a high level of education. Smoking prevalence in the first generation decreased with increasing duration of residence, and the prevalence in the second generation converges towards that of the native-born Germans. This may indicate that Turkish male migrants with a high level of education partly adopt a German life style. In contrast, Turkish male migrants with a low level of education showed a negative generation effect over time, in line with the results of Dill et al. [8]. Within the first generation the smoking prevalence partly converged towards, but also exceeded the smoking prevalence of native-born Germans. Thus, the well-known association between low socioeconomic status and higher risk behaviour is once more confirmed here. But it is also important to note that the second generation group with a low level of education had a higher smoking prevalence than the first generation and the native-born Germans. In contrast to the second-generation Turkish migrants with a high level of education, they do not seem to adopt German smoking behaviour, but rather intensify theirs. This could be interpreted an indication of lack of integration. Together with the lower level of education it may increase the smoking prevalence, perhaps both factors interacting with one another. But it is also possible that migrants adopt to the behaviour of 'their' socioeconomic group in the host country. This could explain our findings in men, but not for those in women.

There is also evidence for a "health transition" among women. Older women in Turkey have a very low smoking prevalence [1,2]. But among Turkish women in Germany, smoking prevalence increases over the generations and even exceeds the prevalence of German women. This may be an indication that cultural attitudes among female Turkish migrants of the second generation differ from those of their parents' generation in this respect. Among the first generation, there is a convergence towards the native-born Germans with increasing duration of residence. This could be the expression of a health transition towards the risk behaviour of the native-born Germans. In contrast to male Turkish migrants, an association between socioeconomic status and smoking is less obvious among female Turkish migrants. The influence of generation and age seem to play a more important role in determining smoking patterns than the level of education.

The fact that with respect to a possible convergence, our results would lead to different interpretations for men and women may also be due to the different stages of the tobacco epidemic. This could, however, not explain why interpretations would also differ between migrants with a high level and those with a low level of education.

The strengths of this study are the large number of respondents included, its representativeness, its use of detailed information on migrant status, rather than the unsatisfactory indicator "nationality", and the stratification of results according to level of education, age and length of residence.

The study also has limitations. Selection bias or response bias cannot entirely be ruled out. For example, language problems might have somewhat reduced the representativeness of the results for the Turkish population in Germany. The questionnaire is, however, also available in Turkish, so even Turkish migrants with little knowledge of the German language can respond. Furthermore, the most frequent method of data collection was the face-to-face interview, such that misunderstandings of questions and language problems should have occurred only rarely.

Further, smoking prevalences might have been underestimated due to incorrect answers. The percentage of proxy interviews was high for youngsters between 15 and 19 (75,0%). It is possible that parents stated the wrong smoking status for their children, or that younger interviewees denied their smoking when filling in the questionnaire together with their parents. Such an underestimation, however, would be a non-differential misclassification, because we failed to find any indication of differences between the Turkish migrants' and the German reference population's response behaviour. Apart from that, such an underestimation seems rather improbable because the study has excluded persons under the age of 18, where denial of smoking might be expected to be more likely.

Responding to the question about smoking status is not obligatory. The non-response rate of 16.0% for this item is higher than the average item non-response rate of 9.0–13.0% [18]. Turkish migrants showed a slightly higher non-response rate for the smoking status in our study, but the difference was small, and thus no evidence that a particular group selectively failed to answer this question.

We used the level of education as a proxy variable for socioeconomic status, although we were aware that this variable cannot fully describe socioeconomic status. Nevertheless it is a common method, and including the income would not increase validity. We dichotomised the variable into high and low level of education because a more differentiated categorisation based only on information about the highest level of education would be problematic.

Regarding the variable 'smoking status', we combined occasional and regular smokers because of the small percentage of occasional smokers. Furthermore, we would argue that our hypothesis can be examined without distinguishing between occasional and regular smokers. The aim of this study was to analyse whether a transition effect can be found in the prevalence of smoking. Thus, stratification for the number of cigarettes smoked would not have added significant information to answer this question. Furthermore, categorisation into 'occasional' and 'regular' was self-reported and not verified by examining a biological marker, thus, it has a high uncertainty and categories can be expected to overlap.

One limitation is the small number of persons in some of the strata after adjustment for age, sex and level of education. Such findings may be subject to random variation and are thus difficult to interpret.

In our analysis, we could not consider possible clustering of smoking behaviour in households. Respondents from one household could be more similar with regard to smoking status than respondents recruited by simple random sampling in the general population. Thus, the p-values we estimated might be slightly too low.

The Microcensus is a cross-sectional data set and does not provide any longitudinal data. Thus, it is of limited usefulness for assessing changes over time. This weakens our analysis of a possible development over the generations and with increasing duration of residence. It seems possible that the observed differences between the generations are due to a cohort effect rather than convergence, and hence, not the expression of a health transition. This would imply that the age cohorts of Turkish migrants in Germany have stable smoking prevalences that reflect the prevalence in Turkey at the time of migration. The presence of a "cohort effect" cannot be excluded in a study with cross-sectional data. But the smoking prevalence in Turkey has been high over a long period of time and decreased only slightly in recent years. This makes a cohort effect an unlikely explanation for our findings.

## Conclusion

Our study shows different trends in smoking prevalences over generations between men and women with a Turkish migration background. It indicates that migration can be conceptualised as a health transition with regard to risk behaviour. Although our data are representative, further studies are needed to analyse the health situation of migrants longitudinally. Our results also indicate a need for preventive action. On the one hand, male Turkish migrants should be supported in reducing smoking even further. On the other hand, there is need for intensified smoking prevention among female Turkish migrants. Prevention efforts need to take into account the different needs of people of different social status or levels of education, and how these combine with the migration background. This should make it possible to reach especially high-risk groups such as Turkish male migrants with a low educational level or Turkish female migrants of the second generation.

## Competing interests

The authors declare that they have no competing interests.

## Authors' contributions

JS, OR and AR conceived the study. JS coordinated the study. AR performed the data analysis and wrote the first draft of the manuscript. JS participated in the data analysis. All authors helped to draft the manuscript and read and approved it in its final form.
